# Recombinant IL-7/HGFβ Hybrid Cytokine Enhances T Cell Recovery in Mice Following Allogeneic Bone Marrow Transplantation

**DOI:** 10.1371/journal.pone.0082998

**Published:** 2013-12-12

**Authors:** Laijun Lai, Mingfeng Zhang, Yinhong Song, Debra Rood

**Affiliations:** 1 Department of Allied Health Sciences, University of Connecticut, Storrs, Connecticut, United States of America; 2 University of Connecticut Stem Cell Institute, University of Connecticut, Storrs, Connecticut, United States of America; Beth Israel Deaconess Medical Center, Harvard Medical School, United States of America

## Abstract

T cell immunodeficiency is a major complication of bone marrow (BM) transplantation (BMT). Therefore, approaches to enhance T cell reconstitution after BMT are required. We have purified a hybrid cytokine, consisting of IL-7 and the β-chain of hepatocyte growth factor (HGFβ) (IL-7/HGFβ), from a unique long-term BM culture system. We have cloned and expressed the IL-7/HGFβ gene in which the IL-7 and HGFβ genes are connected by a flexible linker to generate rIL-7/HGFβ protein. Here, we show that rIL-7/HGFβ treatment enhances thymopoiesis after allogeneic BMT. Although rIL-7 treatment also enhances the number of thymocytes, rIL-7/HGFβ hybrid cytokine was more effective than was rIL-7 and the mechanisms by which rIL-7 and rIL-7/HGFβ increase the numbers of thymocytes are different. rIL-7 enhances the survival of double negative (DN), CD4 and CD8 single positive (SP) thymocytes. In contrast, rIL-7/HGFβ enhances the proliferation of the DN, SP thymocytes, as well as the survival of CD4 and CD8 double positive (DP) thymocytes. rIL-7/HGFβ treatment also increases the numbers of early thymocyte progenitors (ETPs) and thymic epithelial cells (TECs). The enhanced thymic reconstitution in the rIL-7/HGFβ-treated allogeneic BMT recipients results in increased number and functional activities of peripheral T cells. Graft-versus-host-disease (GVHD) is not induced in the rIL-7/HGFβ-treated BMT mice. Therefore, rIL-7/HGFβ may offer a new tool for the prevention and/or treatment of T cell immunodeficiency following BMT.

## Introduction

BMT, the most common cell-based therapy applied today, is widely used in the treatment of cancer, aplastic anemia, and primary and secondary immunodeficiency disorders. Despite improvements in the overall patient survival, transplant recipients often experience prolonged periods of T cell recovery, which contributes to a high risk of infections, and occurrence or relapse of cancers [1-4]. Therefore, approaches to enhance the kinetics of T cell recovery after BMT are required.

The thymus is the primary organ for T cell development. T cell progenitors in the thymus undergo positive and negative selection, generating T cells with a diverse TCR repertoire, able to react with alloantigens, but tolerant to self-antigens. However, the thymus is susceptible to damage from pre-BMT conditioning and GVHD [1-4]. In addition, the thymus undergoes age-dependent involution that progressively decreases its T cell reconstitution ability [5,6].

We have purified a hybrid cytokine, consisting of IL-7 and HGFβ (IL-7/HGFβ), from a unique long-term BM culture system. We have cloned and expressed an IL-7/HGFβ gene in which the IL-7 and HGFβ genes are connected by a flexible linker to generate rIL-7/HGFβ fusion protein [7]. We previously reported that in vivo administration of rIL-7/HGFβ significantly enhances thymopoiesis after syngeneic BMT, resulting in increased numbers of total and naïve T cells in the periphery of the recipients [8]. 

In this study, we investigated whether rIL-7/HGFβ could enhance thymocyte regeneration after allogeneic BMT (allo-BMT), a more clinically relevant model. We show that, although in vivo administration of both rIL-7 and rIL-7/HGFβ significantly increased the numbers of thymocytes, rIL-7/HGFβ hybrid cytokine was ~1.5 times more effective than was rIL-7 alone or together with the individual factor rHGFβ. The mechanisms by which rIL-7 and rIL-7/HGFβ increase the numbers of thymocytes are different. rIL-7 enhances the survival of DN and SP thymocytes by enhancing the expression of Bcl-2, whereas rIL-7/HGFβ induces the proliferation of these cells. rIL-7/HGFβ also enhances the survival of pre-selection DP thymocytes, at least in part, by increasing the expression of Bcl-xL. In addition, rIL-7/HGFβ increases the number of ETPs and TECs. The enhanced thymopoiesis in the rIL-7/HGFβ-treated allo-BMT recipients resulted in increased numbers of T cells in the periphery. Furthermore, the functions of peripheral T cells in the rIL-7/HGFβ-treated allo-BMT recipients were rapidly restored, but GVHD was not induced. Therefore, rIL-7/HGFβ may offer a new approach to preventing and/or correcting T cell deficiency post-BMT.

## Materials and Methods

### Mice

Four- to ten-week-old C57BL/6 (B6), B6.SJL-*Ptprc* ^a^ *Pepc* ^b^/BoyJ (CD45.1^+^ B6), B6C3F1, BALB/c, and B6.129 S2-Tcra^tm1Mom^/J mice were purchased from the Jackson Laboratory. All experimental procedures involving mice were approved by the University of Connecticut Animal Care and Use Committee and were conducted in accordance with NIH guidelines. All efforts were made to minimize animal suffering and discomfort and to reduce the number of animals used.

### BMT Procedure

BM cell suspensions were harvested from mice by flushing the marrow from the femurs and tibias with cold RPMI 1640 (Life Technologies, Grand Island, NY) supplemented with sodium bicarbonate (2 mg/ml) and 1% HEPES (1.5 M). T cell-depleted (TCD) BM cells were prepared by incubating the BM cell suspensions with anti-Thy1.2 antibody for 30 min at 4°C, followed by incubation with low-TOX-M rabbit complement (Cedarlane Laboratories, Hornby, ON, Canada), as described [9]. Recipients received 900-1200 cGy total body irradiation from a 137Cs source (Gammator-50 Gamma Irradiator; Radiation Machinery Corporation, Parsippany, NJ). Two to four hours later, the mice were injected i.v. with 2 X10^6^ TCD-BM. Groups of mice were then injected i.p. with the equimolar amounts of rIL-7/HGFβ (15 μg), rIL-7 (5 μg) and/or rHGFβ (10 μg), or PBS at 2-day intervals from days 1 to 26 after BMT, as described [8]. 

### Flow Cytometry analysis

TECs were isolated by flow cytometry using the protocol of Gray et al [10]. Single cell suspensions of thymocytes and splenic cells were stained with the fluorochrome-conjugated antibodies as described [11]. For intracellular staining, the cells were first permeabilized with a BD Cytofix/Cytoperm solution for 20 minutes at 4°C. Direct or indirect staining of fluorochrome-conjugated antibodies included: CD4, CD8, CD25, CD44, CD62L, c-kit, IL-7Rα, BP-1, CD45, I-A, H-2K^b^, CD45.1, Ki67, CD69, EpCAM1, IL-2, IFN-γ, TNFα, Bcl-2, and a panel of TCR Vβ clonotypes (BioLegend, BD Biosciences, San Jose, CA, or eBioscience, San Diego, CA), as well as Bcl-xL (Cell Signaling Technology, Inc., Danvers, MA). ETPs were identified by phenotypic analysis (Lineage^-^ c-kit^+^ IL-7Rα^-^ CD44^+^CD25^-^) as described [8,11]. An antibody cocktail, composed of antibodies against TER-119, B220, CD19, IgM, Gr-1, CD11b, CD11c, NK1.1, TCRβ, CD3e, and CD8α, was used to identify lineage negative cells. Annexin V and terminal deoxynucleotidyltrasferase dUTP nick end labeling (TUNEL, APO-DIRECT) apoptosis detections kits were purchased from BD Biosciences. The samples were analyzed on a FACSCalibur or LSR II flow cytometer (BD Biosciences). 

### In vitro DP thymocyte survival assay

CD4 and CD8 DP thymocytes were isolated from TCRα mutant mice by immunomagnetic cell separation (Miltenyi Biotec., Auburn, CA). Because the thymocytes of the TCRα mutant mice are largely devoid of CD4 or CD8 SP [12], DP thymocytes were purified by either anti-CD4 or CD8 antibody. Purified DP thymocytes were cultured with equimolar amounts of rIL-7/HGFβ (30 ng/ml), rIL-7 (10 ng/ml) and/or rHGFβ (20 ng/ml), or PBS. Cells were harvested and analyzed for annexin V expression 24 and 48 hour later.

### Western Blot

 Cells were washed with PBS, resuspended in sample buffer, subjected to sodium dodecyl sulfate polyacrylamide gel electrophoresis, and then transferred to Immobilon-P membranes (Millipore, Bedford, MA). The membranes were incubated with rabbit polyclonal antibody against mouse Bcl-xL (Cell Signaling Technology), washed, incubated with HRP-linked secondary antibody against rabbit IgG, and then developed with enhanced chemiluminescence (GE Healthcare Biosciences, Pittsburgh, PA).

### BrdU labeling

Mice were injected i.p. with 2 doses of BrdU (Sigma-Aldrich, St Louis, MO) at 1 mg/dose at 2-hour intervals [13]. Cells were isolated 2 hours following the second dose and assessed for BrdU incorporation by BrdU flow kit (BD Biosciences).

### MLRs

Splenocytes (normalized to 1 X 10^5^ T cells/well) from BMT recipients were cultured in the presence or absence of irradiated (2,000 cGy) splenocytes (2 X 10^5^ cells/well) from different mouse strains in a 96-well plate for 4 days. Cell proliferation was measured by BrdU Labeling and Detection Kit III (Roche Allied Science, Mannheim, Germany) according to the manufacturer's instructions. Absorbance (measured as OD), proportional to Brdu uptake, was measured at 405 nm using an ELISA microplate reader (BioTek, Winooski, VT). The data are expressed as stimulation index (OD in MLRs/OD in spontaneous proliferation). 

### Anti-CD3 and anti-CD28 T cell proliferation assay

Splenic T cells were stimulated with plate-bound anti-CD3 (clone 145-2c 11) and anti-CD28 (clone 37.51) at 5 μg/ml and cultured for 4 days. Cell proliferation was measured by BrdU Labeling and Detection Kit III as described above. The stimulation index was calculated as the ratio of the OD from stimulated cells over the OD from unstimulated cells.

### Assessment of GVHD

The severity of GVHD was evalulated with a clinical GVHD scoring system as described [11,14-16]. In brief, BMT recipients in coded cages were individually scored every week for five clinical parameters on a scale from 0 to 2: weight loss, posture, activity, fur texture and skin integrity. A clinical GVHD index was generated by summation of the five criteria scores (maximum index = 10). Animals were euthanized 75 days after BMT, and GVHD target organs (small bowel, large bowel and liver) were harvested for histopathological analysis. Briefly, organs were formalin-preserved, paraffin-embedded, sectioned and hematoxylin/eosin-stained. A semiquantitative score consisting of 19 to 22 different parameters associated with GVHD was calculated, as described [11,14-16]. 

### Statistical analysis

P-values were based on two-sided Student’s test. A confidence level above 95% (p<0.05) was determined to be significant.

## Results

### rIL-7/HGFβ treatment enhances thymopoiesis after allo-BMT

**Figure 1:**
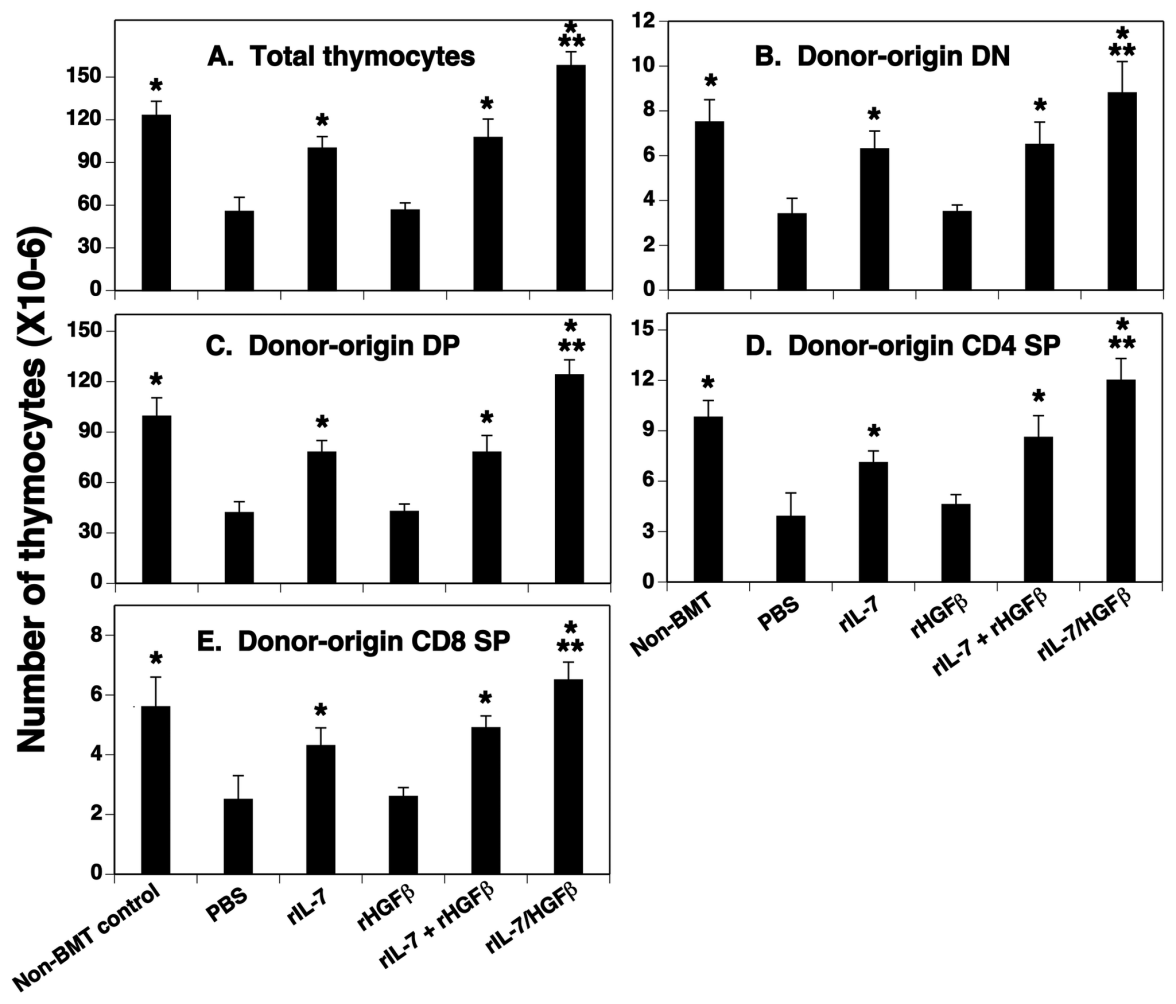
The number of thymocyte subsets was significantly increased in allo-BMT recipients after rIL-7/HGFβ treatment. Lethally irradiated mice (BALB/c, 4-10 week old) were injected i.v. with 2 X10^6^ TCD-BM from B6 mice. Groups of mice were then injected i.p. with the equimolar amounts of rIL-7/HGFβ (15 μg), rIL-7 (5 μg) and/or rHGFβ (10 μg), or PBS at 2-day intervals from days 1 to 26 after BMT. The number of (A) total thymocytes, donor-origin (B) CD4 and CD8 DN, (C) CD4 and CD8 DP, (D) CD4 SP, and (E) CD8 SP thymocytes was analyzed on day 30 after BMT. Means + S.D. are presented. The data are representative of 2 independent experiments with 4-6 mice per group. * P<0.05 compared with PBS-treated mice. ** P<0.05 compared with rIL-7 and/or rHGFβ-treated mice.

We previously reported that *in vivo* administration of optimal equimolar rIL-7/HGFβ (15µg), rIL-7 (5µg), or the combination of individual factors rIL-7 (5µg) and rHGFβ (10µg) significantly enhanced thymopoiesis in syngeneic BMT recipients [8]. In that study, rIL-7/HGFβ was more effective at enhancing thymic cellularity than was rIL-7 alone or together with rHGFβ. To determine whether this would also occur in allo-BMT recipients, lethally irradiated BALB/c mice (H-2^d^) were injected i.v. with TCD BM from B6 mice (H-2^b^). The numbers of total thymocytes and donor-origin thymocyte subsets were analyzed one month later. Similar to the results from the syngeneic BMT recipients [8], the number of thymocytes was markedly increased in rIL-7/HGFβ-treated allo-BMT mice, and all thymocyte subsets were affected proportionately (Figure 1). Treatment with rIL-7 alone or together with rHGFβ also significantly increased the number of thymocytes. However, the number of thymocytes in rIL-7/HGFβ-treated mice was about 1.5-fold over rIL-7-, or rIL-7 and rHGFβ-treated mice. The increased number of thymocytes was maintained through at least day 75 (Figure S1A). We also evaluated the effect of rIL-7/HGFβ on thymocyte reconstitution in a parent-F1 model (CD45.1^+^ B6 → B6C3F1) and again found a significant increase in the number of thymocytes (Figure S2). Taken together, these data suggest that rIL-7/HGFβ treatment can rapidly and durably enhance thymocyte reconstitution following allo-BMT. 

### rIL-7/HGFβ enhances the survival of pre-selection DP thymocytes and induces the proliferation of DN and SP thymocytes

**Figure 2:**
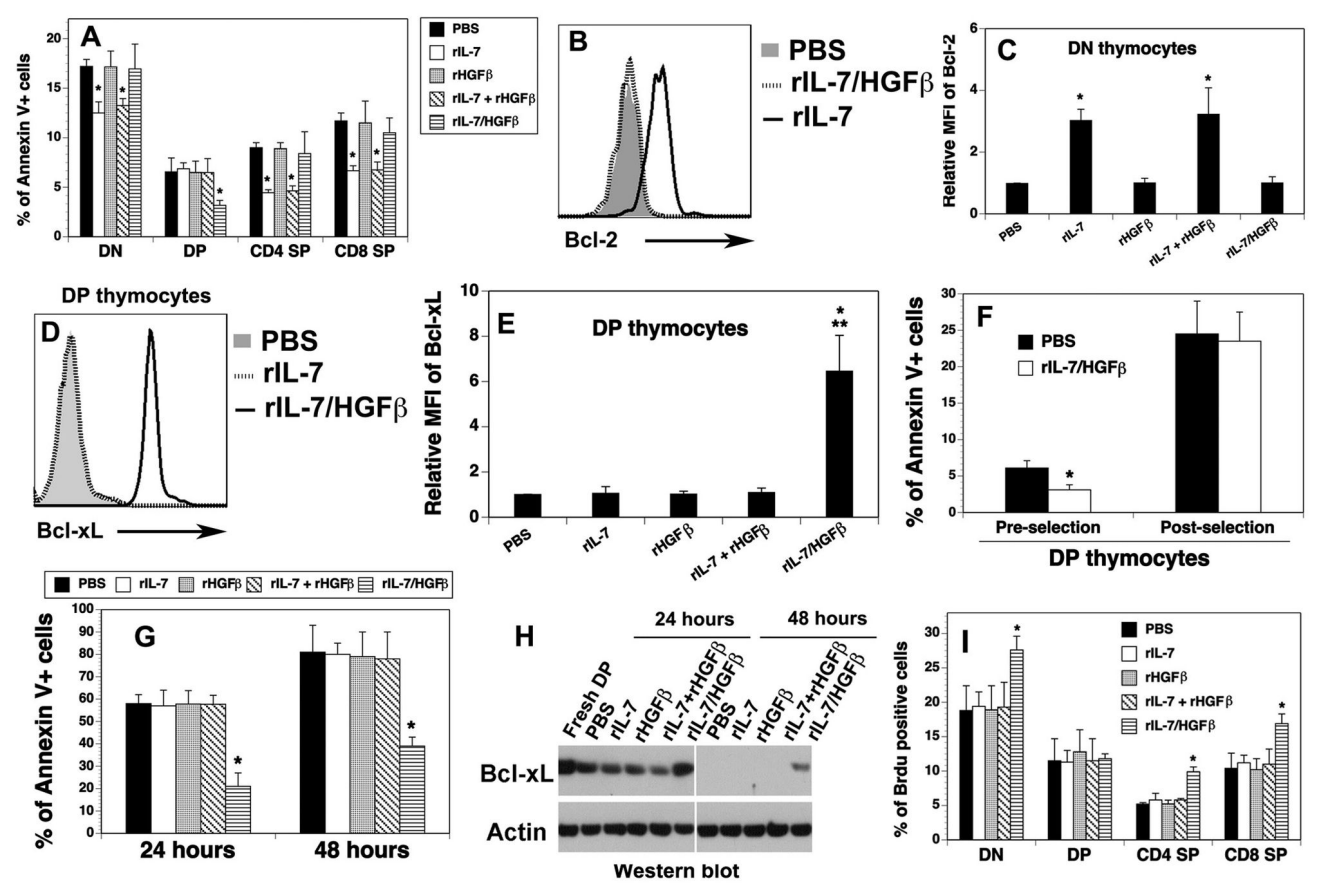
The survival of pre-selection DP and the proliferation of DN and SP thymocytes were enhanced by rIL-7/HGFβ. (A-F, I) Lethally irradiated BALB/c mice were injected with TCD-BM from B6 mice and treated with cytokines as in Figure 1. On day 30 after BMT, (A) The percentages of Annexin V^+^ cells in each thymocyte subset were analyzed by flow cytometry. (B) Representative histograms of intracellular staining for Bcl-2 by DN thymocytes. (C) Relative mean fluorescence intensity (MFI) + SD of Bcl-2 by DN thymocytes from the cytokine-treated BMT mice. (D) Representative histograms of intracellular staining for Bcl-xL by DP thymocytes. (E) Relative MFI of Bcl-xL by DP thymocytes. (F) rIL-7/HGFβ treatment enhances the survival of CD69^-^ pre-selection DP thymocytes. (G, H) rIL-7/HGFβ maintains the survival and the expression of Bcl-xL of DP thymocytes *in* *vitro*. Purified DP thymocytes were cultured in the presence of equimolar amounts of rIL-7/HGFβ (30 ng/ml), rIL-7 (10 ng/ml) and/or rHGFβ (20 ng/ml), or PBS for 24 and 48 hours. Cells were analyzed for (G) annexin V^+^ cells by flow cytometry and (H) the expression of Bcl-xL by Western blot. (I) the cytokine-treated allo-BMT mice were injected i.p. with BrdU. The percentages of BrdU^+^ cells by thymocyte subsets were then analyzed by flow cytometry 4 hour later. The data are representative of 2 independent experiments with 4-6 mice per group. * P<0.05 compared with PBS-treated mice.

To gain insight into the mechanisms by which rIL-7/HGFβ enhances thymopoiesis, we examined cell survival of thymocytes from the cytokine-treated mice by analyzing annexin V^+^ apoptotic cells. We found that the percentage of apoptotic cells in DP, but not in DN, as well as CD4 and CD8 SP thymocyte subsets, was decreased in the rIL-7/HGFβ-treated mice, as compared to the PBS-treated mice (Figure 2A). In contrast, the percentages of apoptotic cells in DN, as well as CD4 and CD8 SP, but not DP thymocyte subsets, were significantly decreased in the rIL-7-, or rIL-7 and rHGFβ-treated mice (Figure 2A). By using a TUNEL apoptosis detection assay, we observed an identical pattern of decreased apoptotic cells in DP thymocytes in rIL-7/HGFβ-treated mice, and of decreased apoptotic cells in DN and SP thymocytes in rIL-7-, or rIL-7 plus rHGFβ-treated mice (data not shown). 

Because Bcl-2 has been reported to play a critical role in rIL-7-mediated DN thymocyte survival [17], we analyzed Bcl-2 expression by these cells. We found that rIL-7, or rIL-7 plus rHGFβ significantly enhanced the expression of Bcl-2 protein in DN and SP thymocytes (Figure 2B, 2C and data not shown). In contrast, rIL-7/HGFβ treatment did not enhance the expression of Bcl-2 in these cells, as compared to PBS treatment. 

Another anti-apoptotic molecule, Bcl-xL, has been shown to play a critical role in the survival of DP thymocytes [18,19]. We then examined the expression of Bcl-xL by DP thymocytes from the cytokine-treated BMT mice. As shown in Figure 2D and 2E, rIL-7/HGFβ treatment substantially up-regulated the expression of Bcl-xL protein, whereas rIL-7 and/or rHGFβ did not. The results are consistent with the enhanced survival of DP thymocytes by rIL-7/HGFβ, but not by rIL-7 and/or rHGFβ. 

The DP compartment can be divided into CD69^-^ pre-selection and CD69^+^ post-selection subsets [19-22]. We then determined whether rIL-7/HGFβ increased the survival of pre-selection and/or post-selection DP thymocytes. Consistent with previous reports [19-22], we found that 83-89% and 11-17% of DP thymocytes were CD69^-^ pre-selection and CD69^+^ post-selection, respectively (data not shown). As shown in Figure 2F, rIL-7/HGFβ significantly decreased the apoptosis of CD69^-^ pre-selection DP thymocytes, but not that of post-selection DP thymocytes. To confirm that rIL-7/HGFβ affects pre-selection DP thymocytes, DP thymocytes from TCRα^-/-^ mice that fail to undergo positive selection were used to examine whether rIL-7/HGFβ can inhibit the spontaneous apoptosis of these cells *in vitro*. To this end, DP thymocytes were purified from TCRα^-/-^ mice and cultured in the presence of equimolar amounts of rIL-7/HGFβ, rIL-7 and/or rHGFβ, or PBS. The percentages of Annexin V^+^ apoptotic cells were then analyzed 24 and 48 hours later. As shown in Figure 2G, about 58% of DP thymocytes underwent apoptosis in the presence of PBS, rIL-7 and/or rHGFβ after 24-hour cultures, while only 20% of the cells underwent apoptosis in the rIL-7/HGFβ containing cultures. At 48 hours, 78-82% thymocytes were annexin V^+^ cells in the absence of rIL-7/HGFβ, whereas only 39 % of DP bound annexin V in the presence of rIL-7/HGFβ. These results further confirm our *in vivo* data that rIL-7/HGFβ enhances the survival of pre-selection DP thymocytes. Furthermore, Western blot analysis showed that, after 24-hours in cultures, the expression of Bcl-xL protein in DP thymocytes was markedly reduced in the presence of PBS, rIL-7 and/or rHGFβ, whereas Bcl-xL expression in the presence of rIL-7/HGFβ was comparable to that in freshly harvested DP thymocytes (Figure 2H). At 48 hours, Bcl-xL expression in the DP thymocytes was undetectable in the absence of rIL-7/HGFβ, but still detectable in the presence of rIL-7/HGFβ (Figure 2H). The data suggest that rIL-7/HGFβ enhanced the survival of pre-selection DP thymocytes, at least in part, by increasing the expression of Bcl-xL. 

Because the enhanced thymopoiesis in the cytokine-treated allo-BMT mice could also be due to enhanced cell expansion, we examined the proliferation of thymocyte subsets from the cytokine-treated BMT mice by *in vivo* Brdu labeling. As shown in Figure 2I, rIL-7/HGFβ treatment significantly increased the percentages of BrdU^+^ cells in DN, as well as CD4 and CD8 SP, but not DP thymocyte subsets, whereas rIL-7, or rIL-7 plus rHGFβ did not significantly increase the percentages of BrdU^+^ cells in any thymocyte subset. An identical pattern was obtained when thymocyte proliferation was determined by flow cytometric analysis of Ki67 protein expression (data not shown). 

Taken together, our results suggest that rIL-7 enhances the survival of DN and SP thymocytes, whereas rIL-7/HGFβ enhances the survival of pre-selection DP, and the proliferation of DN and SP thymocytes. The enhanced survival of DP by rIL-7/HGFβ is likely due, at least in part, to the increased expression of BCL-xl. 

### rIL-7/HGFβ treatment enhances the numbers of ETPs and TECs after allo-BMT

**Figure 3:**
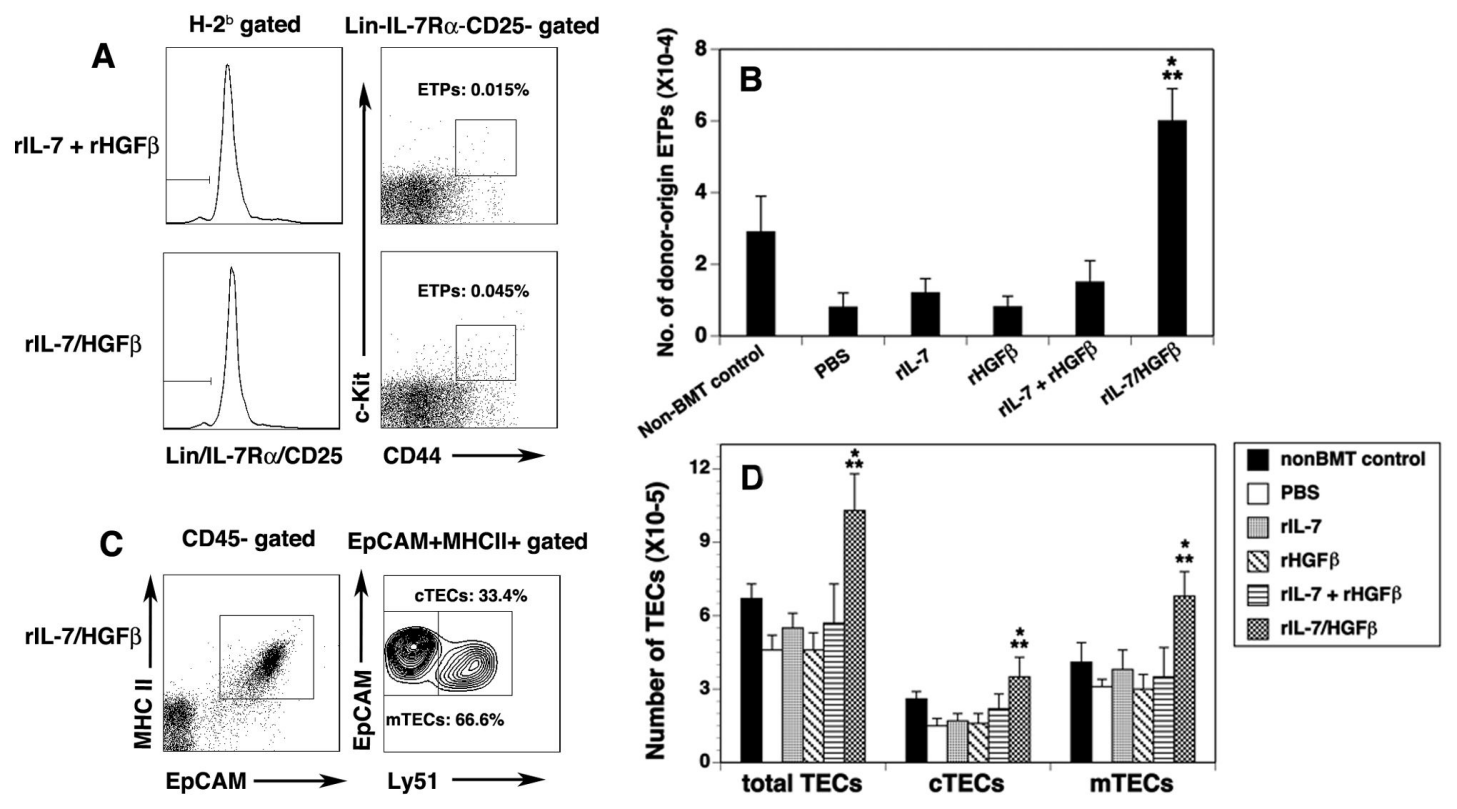
The number of ETPs and TECs was significantly increased in allo-BMT recipients after rIL-7/HGFβ treatment. Lethally irradiated BALB/c mice were injected with TCD-BM from B6 mice and treated with cytokines as in Figure 1. On day 30 after BMT, ETPs and TECs were analyzed. (A) Representative flow cytometric profiles showing the percentage of donor-origin lineage^-^ c-kit^+^ IL-7Rα^-^ CD44^+^CD25^-^ ETPs in total thymocytes. (B) Number of donor-origin ETPs in the cytokine-treated BMT mice. (C) Representative flow cytometric profiles showing the percentage of CD45^-^EpCAM^+^MHC II^+^Ly51^+^ cTECs and CD45^-^EpCAM^+^MHC II^+^Ly51^-^ mTECs in total TECs of the rIL-7/HGFβ-treated BMT mice. (D) Number of total TECs, cTEC and mTECs in the cytokine-treated BMT mice. The data are representative of 2 independent experiments with 4-6 mice per group. * P<0.05 compared with PBS-treated mice. ** P<0.05 compared with rIL-7 and/or rHGFβ-treated mice.

We previously reported [8] that, in syngeneic BMT recipients, rIL-7/HGFβ treatment significantly enhanced the numbers of ETPs that contain canonical thymocyte precursors [23-25]. We wanted to determine whether this also occurred in the allo-BMT recipients. Indeed, rIL-7/HGFβ significantly increased the numbers of ETPs by 5-9-fold over those in the PBS, rIL-7 and/or rHGFβ-treated mice, and about 2-fold over those in non-BMT control mice on day 30 after BMT (Figure 3A). The increase in ETP number was maintained for at least 75 days (Figure S1B). Because T cell development in the thymus depends not only on the availability of ETPs, but also on the thymic microenvironment, of which TECs are the major component [26,27], we then analyzed TEC numbers. rIL-7/HGFβ treatment did not significantly increase the percentage of total TECs (CD45^-^ EpCAM^+^MHC II^+^), cortical (c) TECs (CD45^-^EpCAM^+^MHC II^+^Ly51^+^) and medullary (m) TECs (CD45^-^EpCAM^+^MHC II^+^Ly51^-^), as compared with rIL-7 + rHGFβ treatment (Figure 3C and data not shown). However, because rIL-7/HGFβ significantly increased the number of total cells in the thymus, the number of total TECs, cTECs and mTECs in the rIL-7/HGFβ-treated mice was significantly increased (Figure 3D). In contrast, rIL-7 and/or HGFβ did not increase the number of ETPs and TECs in the allo-BMT recipients. 

### rIL-7/HGFβ treatment results in an increased number of T cells in the periphery after allo-BMT

**Figure 4:**
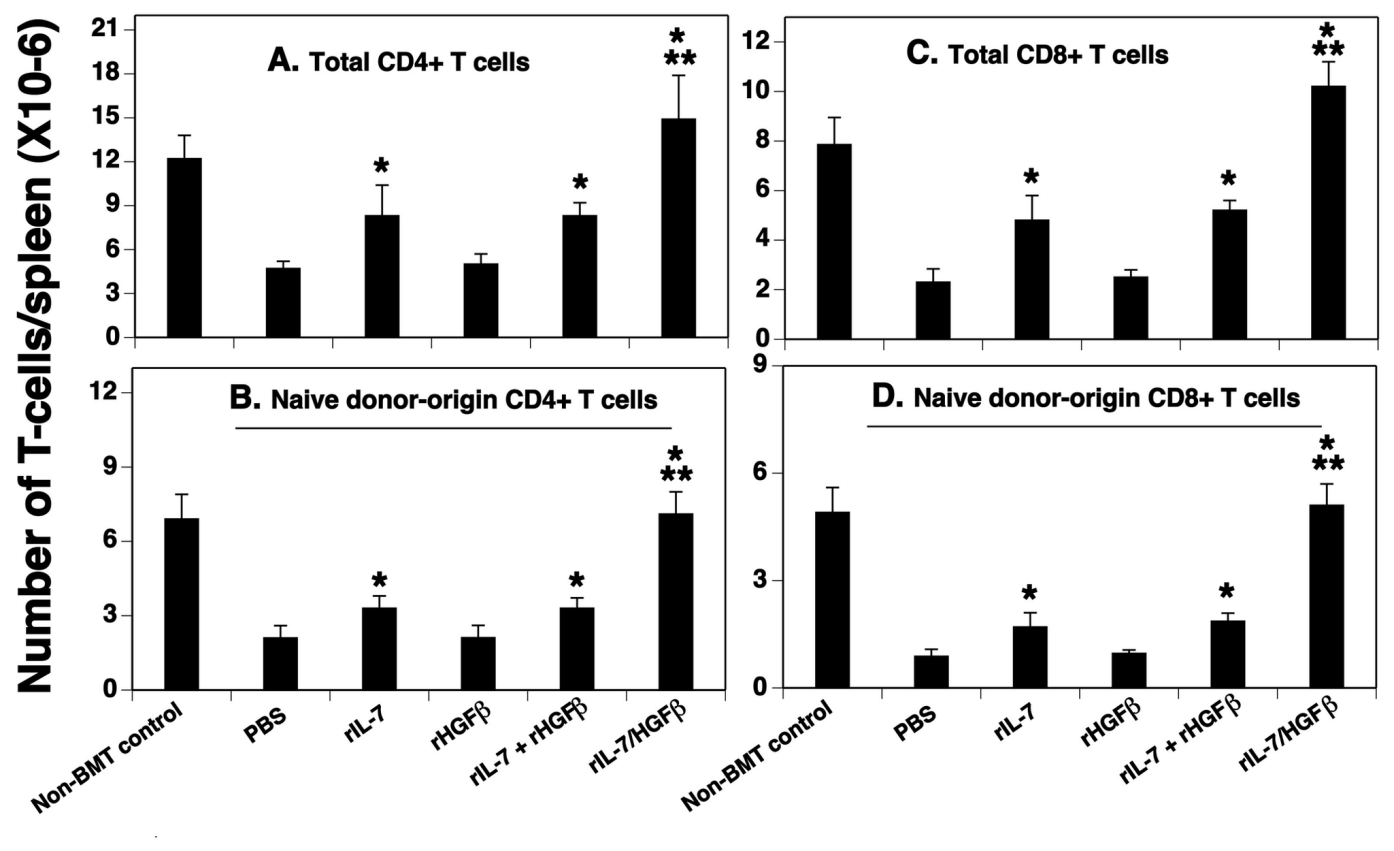
The number of total and naïve T cells in the spleen of allo-BMT recipients was significantly increased after rIL-7/HGFβ treatment. Lethally irradiated BALB/c mice were injected with TCD-BM from B6 mice and treated with cytokines as in Figure 1. On day 30 after BMT, the number of total (A) CD4 and (C) CD8 splenic T cells; and donor-origin naive (CD62L^hi^ CD44^lo^) (B) CD4 and (D) CD8^+^ splenic T cells was evaluated by flow cytometry. The data are representative of 2 independent experiments with 4-6 mice per group. * P<0.05 compared with PBS-treated mice. ** P<0.05 compared with rIL-7 and/or rHGFβ-treated mice.

To determine whether the enhanced thymopoiesis in rIL-7/HGFβ-treated BMT mice could translate into an increased number of peripheral T cells, we examined the number of total and naïve CD4^+^ and CD8^+^ T cells in the spleen. As shown in Figure 4A and C, rIL-7/HGFβ treatment increased the number of total CD4^+^ and CD8^+^ T cells to levels above those observed in non-BMT control mice, and approximately 3-4-fold higher than those observed in mice treated with PBS, whereas rIL-7 alone or together with rHGFβ increased the number by about 2-fold over PBS treatment. rIL-7/HGFβ also increased the number of donor-origin naïve (CD62L^hi^CD44^lo^) CD4^+^ and CD8^+^ T cells to the levels of naïve T cells in non-BMT control mice, and approximately 3-5-fold above those observed in mice treated with PBS, whereas rIL-7 alone or together with rHGFβ increased the number by 1.5-2-fold over PBS treatment (Figure 4B and 4D). By using the method of intrathymical injection of biotin and then determination of biotin-labeled T cells in the spleen 24 hours later [11], we found that the number of biotin-labeled T cells in the rIL-7/HGFβ-treated BMT mice was 3-5-fold higher than that in the PBS-treated mice, and about 2-fold higher than that in the rIL-7, or rIL-7 and rHGFβ-treated mice (data not shown). Taken together, these results suggest that enhanced thymopoiesis in rIL-7/HGFβ-treated BMT mice resulted in enhanced thymic output, leading to increased numbers of T cells in the periphery. 

### Peripheral T cells in rIL-7/HGFβ-treated allo-BMT recipients are functional

**Figure 5:**
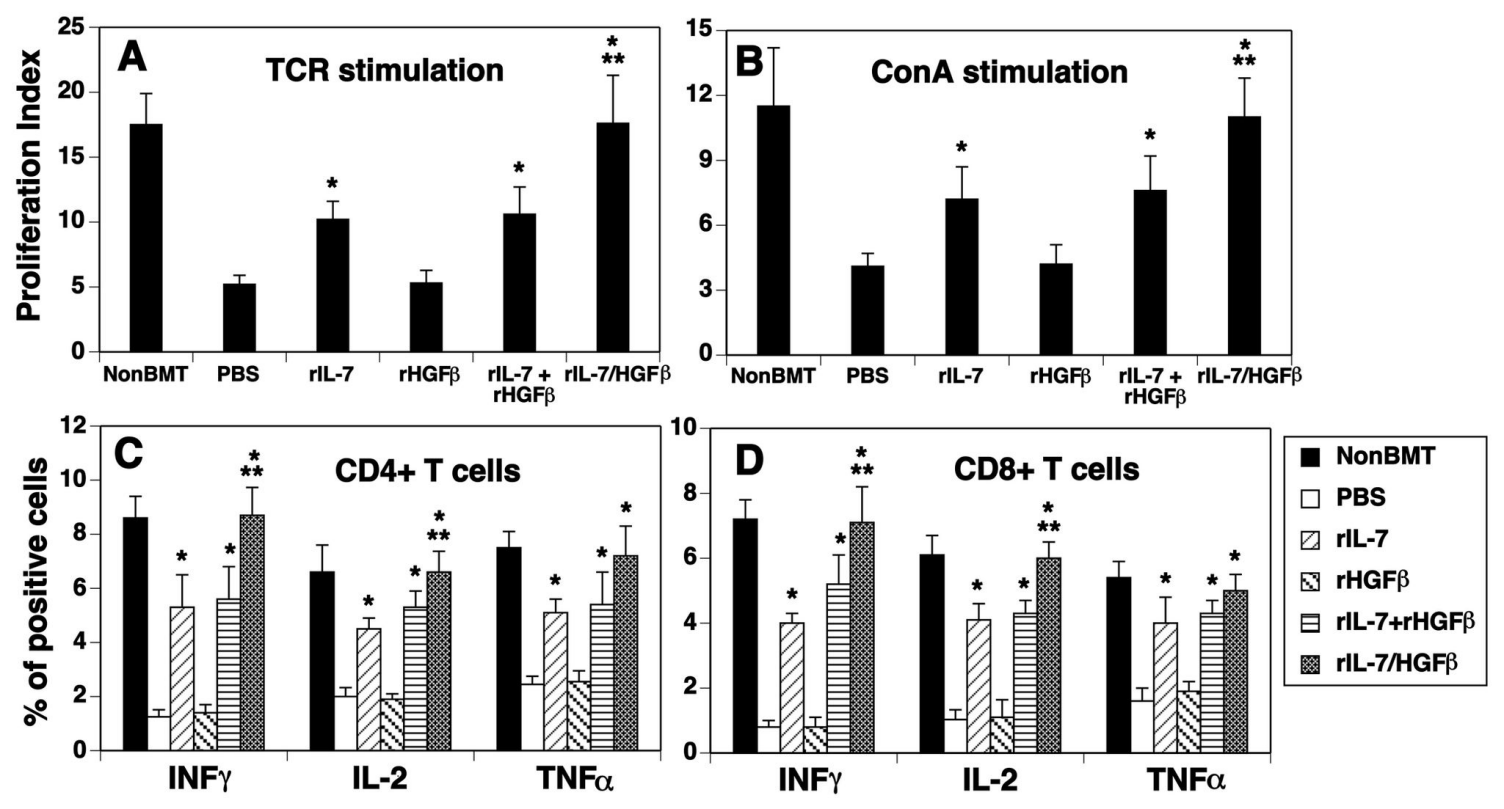
Peripheral T cells in the rIL-7/HGFβ-treated allo-BMT recipients are functional. Lethally irradiated BALB/c mice were injected with TCD-BM from B6 mice and treated with cytokines as in Figure 1. On day 30 after BMT, (A) splenic CD3^+^ T cells were stimulated with anti-CD3 and anti-CD28 antibodies (5 μg/ml), and cell proliferation was determined by BrdU incorporation 4 days later. (B) splenic CD3^+^ T cells were stimulated with Con A (4 μg/ml) and cell proliferation was determined by BrdU incorporation 4 days later. (A and B) Data are shown as stimulation index. (C and D) Splenocytes were stimulated with phorbol myristate acetate and ionomycin, and stained with antibodies for cell surface markers and intercellular cytokines (H-2^**b**^, CD4, CD8, IL-2, IFN-γ, and TNFα). The percentage of IL-2, IFN-γ and TNFα positive cells in donor-origin CD4^+^ and CD8^+^ T cells was determined by flow cytometry. The data are representative of 2 independent experiments with 4-6 mice per group. * P<0.05 compared with PBS-treated mice. ** P<0.05 compared with rIL-7 and/or rHGFβ-treated mice.

We then assessed whether T cells in the rIL-7/HGFβ-treated BMT mice were functional. We first examined T cell proliferation in response to TCR stimulation. As shown in Figure 5A, 1 month after BMT, splenic T cells from PBS-treated mice were significantly less responsive to anti-CD3 plus CD28 stimulations, as compared to normal non-BMT control mice. T cells from the rIL-7-treated mice had higher proliferative capacity than T cells from the PBS-treated mice, but did not reach the levels observed in the normal non-BMT control mice. In contrast, T cells from rIL-7/HGFβ-treated mice on a per cell basis responded significantly better than T cells in the PBS, or rIL-7 and/or HGFβ-treated mice, and almost as well as T cells from the normal non-BMT control mice. A similar pattern was obtained when the splenic T cells were stimulated with Concanavalin A (Con A) (Figure 5B). We then analyzed cytokine-producing cells and found that a significantly greater fraction of CD4^+^ and CD8^+^ T cells from rIL-7/HGFβ-treated BMT mice was able to produce IFN-γ, IL-2 and TNFα after phorbol myristate acetate and ionomycin stimulation, as compared to those from PBS-treated BMT mice (Figure 5C and 5D). Taken together, our results suggest that the number and function of T cells in rIL-7/HGFβ-treated mice are rapidly restored following allo-BMT. 

In addition, we evaluated the TCR repertoire of donor-origin CD4^+^ and CD8^+^ T cells in the spleen of the rIL-7/HGFβ-treated mice. Consistent with our previous data from rIL-7/HGFβ-treated syngeneic BMT recipients [8], we found that these T cells had a diverse TCR repertoire (Figure S3). 

### GVHD in rIL-7/HGFβ-treated allo-BMT recipients is not induced

**Figure 6:**
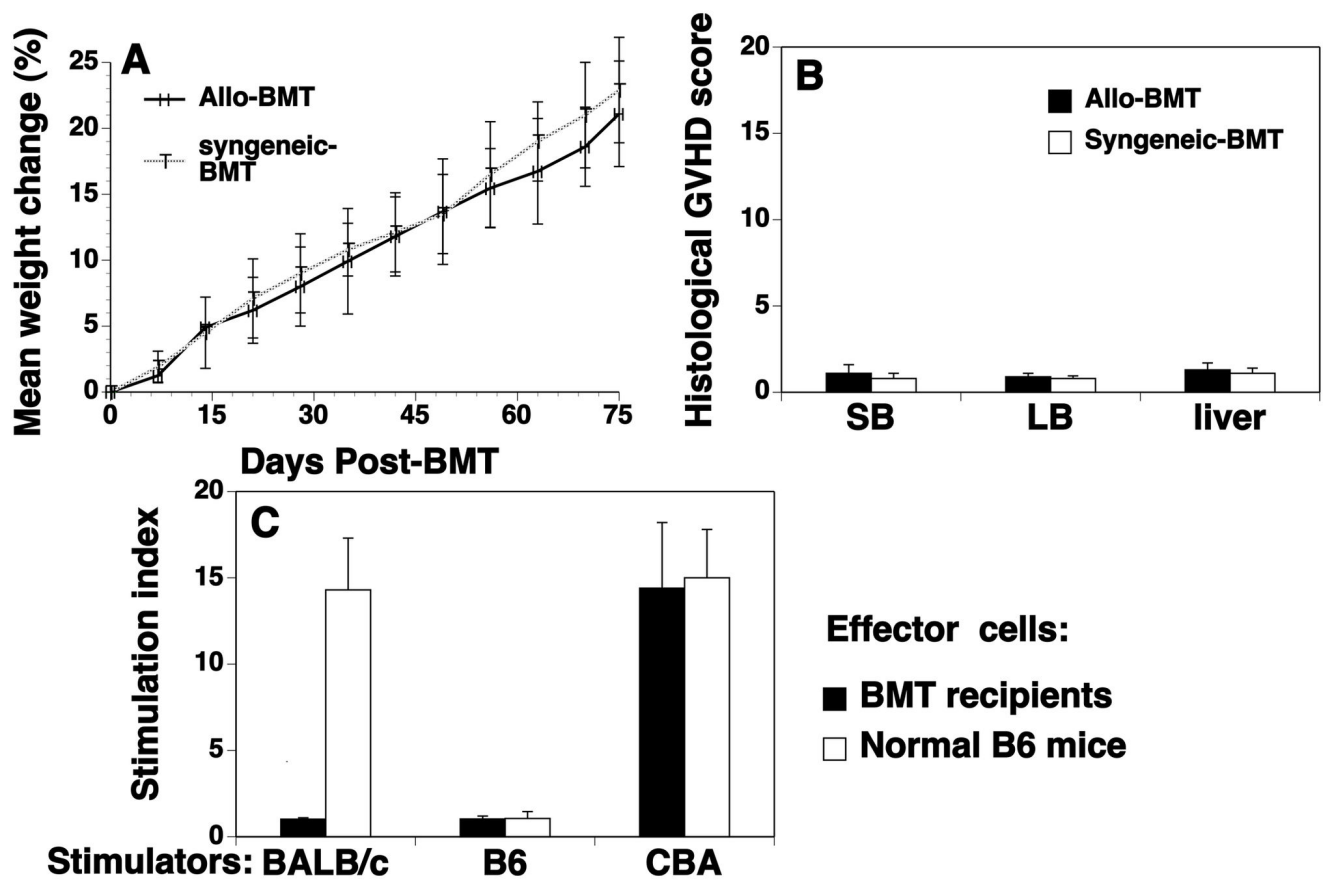
rIL-7/HGFβ-treated allo-BMT recipients do not develop GVHD. Lethally irradiated BALB/c mice were injected with TCD-BM from B6 mice. Syngeneic BMT (both BM and recipients from B6 mice) were used as controls. Both syngeneic and allo-BMT recipients were treated with rIL-7/HGFβ (15 μg) or PBS at 2-day intervals from days 1 to 26 after BMT. (A) weights and (B) histopathological analysis of signs of GVHD in liver, small bowel (SB) and large bowel (LB) were conducted on day 75 after BMT. (C) Splenocytes harvested from the allo-BMT recipients were used as effector cells for MLRs. Splenocytes from normal non-BMT B6 mice were used as controls. The effector cells were cultured with irradiated splenocytes (as stimulators) from normal non-BMT BALB/c, B6, and CBA mice, respectively. Cell proliferation was determined by BrdU incorporation. Mean OD in MLRs/OD in spontaneous proliferation with splenocytes from all-BMT recipients or normal B6 mice as effectors and splenocytes from BALB/c, B6, and CBA mice as stimulators were 0.21/0.20, 0.20/0.20, 2.97/0.21, as well as 2.95/0.21, 0.21/0.20, and 3.15/0.22, respectively. Data are shown as stimulation index. (A-C) The data are representative of 2 independent experiments of 5 mice each group.

GVHD is a serious clinical problem after allo-BMT and remains the major limitation of allo-BMT. We assessed whether GVHD occurred in rIL-7/HGFβ treated allo-BMT recipients. rIL-7/HGFβ treated syngeneic BMT was used as controls. As shown in Figure 6A and 6B, we did not observe any signs of GVHD in rIL-7/HGFβ-treated BMT recipients as determined weight loss and subclinical histopathological analysis. We also observed that both groups of allogeneic and syngeneic BMT recipients survived through at least day 75, and had a clinical GVHD score of 0. By using MLRs to evaluate T cell response to self antigens and alloantigens, we found that splenic T cells from rIL-7/HGFβ-treated BMT mice failed to proliferate in response to stimulation with donor- and host-antigens, but mounted an immune response to alloantigens (Figure 6C). Taken together, our results suggest that the precursors of these newly-formed T cells had undergone normal negative selection in the thymus of the allo-BMT recipients, leading to immune tolerance to the donor and host antigens. 

## Discussion

We previously reported that rIL-7/HGFβ treatment significantly enhanced thymopoiesis after syngeneic BMT in mice [8]. In this study, we have moved into more clinically relevant models, allogeneic BMT. We show here that rIL-7/HGFβ treatment significantly enhances thymopoiesis in allo-BMT recipients, resulting in increased number and functional activities of T cells in the periphery. Although rIL-7 treatment also increases the number of thymocytes, rIL-7/HGFβ is more effective than was rIL-7 alone or together with rHGFβ. The mechanisms by which rIL-7 and rIL-7/HGFβ enhance thymopoiesis are different. rIL-7 enhances the survival of DN and SP thymocytes, whereas rIL-7/HGFβ enhances the survival of DP thymocytes, and the proliferation of DN, CD4 and CD8 SP thymocytes. rIL-7/HGFβ treatment also significantly increases the numbers of ETPs and TECs, whereas rIL-7 does not.

Our data shows that rIL-7 treatment enhances the survival, but not the proliferation of DN and SP thymocytes, consistent with previous reports that IL-7 has a striking effect in sustaining their survival without notable cell division [17,28-30]. Our data that rIL-7 treatment increased the expression of Bcl-2 by DN thymocytes is also consistent with previous reports that Bcl-2 plays a critical role in the IL-7-induced survival of thymocytes [17,28]. Surprisingly, rIL-7/HGFβ treatment did not enhance the survival and the expression of Bcl-2 by DN and SP thymocytes. Instead, rIL-7/HGFβ induced the proliferation of these cells. The mechanism of the different effects of rIL-7 and rIL-7/HGFβ on DN and SP remains unclear. We previously reported that rIL-7/HGFβ stimulated the proliferation of early B-lineage cells by cross-linking IL-7Rα and the HGF receptor c-Met on the cell surface, which results in signal cross-talk downstream of the receptors, leading to novel functional readouts [7]. We have also shown that DN and SP thymocytes express IL-7Rα and c-Met and that rIL-7/HGFβ cross-linked the receptors on DN thymocytes [8]. It is likely that similar receptor cross-linking leads to cell proliferation in the DN and SP thymocytes, whereas the activation of the IL-7Rα by rIL-7 alone results in enhanced survival of thymocytes by Bcl-2. 

Apoptosis in DP thymocytes can occur through αβTCR independent death by neglect and αβTCR dependent negative selection [18,19,31]. We observed that rIL-7/HGFβ enhanced the survival of pre-selection DP thymocytes through an αβTCR independent manner. The lifespan of DP thymocytes is important for normal T cell development [18,19,31]. In normal mice, DP thymocytes are able to initiate multiple, sequential rearrangements at the TCRα locus. A longer survival window of DP thymocytes increases the opportunity for generating a diverse T cell repertoire [18,19,31]. Several studies have shown that Bcl-xL expression in pre-selection DP is critical for the survival of these cells [18,19]. In this study, we show that rIL-7/HGFβ enhances the expression of Bcl-xL and the survival of pre-selection of DP thymocytes in vivo and in vitro. We have previously shown that DP thymocytes express c-Met, but not IL-7Rα [8]. Therefore, the rIL-7/HGFβ-induced expression of Bcl-xL and survival of DP thymocytes may be mediated by the activation of c-Met. However, rHGFβ alone or in combination with rIL-7 did not enhance the expression of Bcl-xL and the survival of DP thymocytes. It is possible that rIL-7/HGFβ cross-linked c-Met and another IL-7 receptor component, γc, on DP thymocytes, which results in signal cross-talk downstream of the receptors and increased expression of Bcl-xL, leading to enhanced survival of DP thymocytes. 

The signaling components connecting the rIL-7/HGFβ signal with Bcl-xL induction remains to be determined. RORγt, TCF-1, c-Myb and HEB have been shown to upregulate the expression of Bcl-xL in DP thymocytes [18,19]. Interestingly, rIL-7 alone inhibits the expression of TCF-1, LEF-1, and RORγt in thymocytes [32]. NF-қB and NFAT4 have also been shown to regulate Bcl-xL expression [33,34], and Egr3 negatively regulates the survival of DP thymocytes [35]. It will be of interest to investigate whether rIL-7/HGFβ regulates these molecules, leading to enhanced expression of Bcl-xL in DP thymocytes. 

We also observed that rIL-7/HGFβ treatment increased the number of ETPs and TECs, whereas individual factor rIL-7 and/or rHGFβ did not. Because both ETPs and TECs do not express the IL-7Rα chain [8], it is possible that rIL-7/HGFβ cross-links c-Met and the γc chain or other receptor(r), such as c-kit [36], on these cells, leading to increased survival/proliferation of ETPs and TECs. It is also possible that the increased number of TECs is secondary to their interactions with regenerating thymocytes, and the increased number of ETPs is due to enhanced generation and migration of their precursors in/from the BM. The latter is supported by our recent observations that rIL-7/HGFβ treatment significantly increased the number of hematopoietic stem cells in the BM [37].

 Our findings have also shown that the enhanced thymopoiesis in the rIL-7/HGFβ-treated BMT recipients results in an increased number of total and naïve T cells in the periphery. The functions of the T cells were rapidly restored after rIL-7/HGFβ treatment, as compared to PBS, rIL-7 and/or rHGFβ treatments. In addition, donor-origin T cells in the recipients had a diverse TCR repertoire in the rIL-7/HGFβ-treated mice. Furthermore, these T cells were immune tolerant to the donor- and host-antigens, but were able to mount an immune response to foreign antigens. GVHD was not induced in the rIL-7/HGFβ-treated allo-BMT recipients. Taken together, these data suggest that the developing thymocytes in the rIL-7/HGFβ-treated allo-BMT mice have undergone normal positive and negative selection. However, although GVHD was not induced in our current T cell depleted BMT model, it will be important to determine whether rIL-7/HGFβ hybrid cytokine affects the development of GVHD in a T cell replete allogeneic setting [38,39], which is underway in our laboratory. 

Because rIL-7 and rIL-7/HGFβ enhance thymopoiesis by different mechanisms, i.e. rIL-7 enhances the survival of DN and SP thymocytes, whereas rIL-7/HGFβ enhances the survival of DP thymocytes and the proliferation of DN and SP thymocytes, we expect that the combination of rIL-7 and rIL-7/HGFβ will synergistically enhance thymopoiesis after BMT, which will be addressed in future studies. 

In summary, rIL-7/HGFβ, when used alone or combined with other factor(s), has potential clinical applications in preventing and/or reversing post-BMT T cell deficiency and its complications.

## Supporting Information

Figure S1 **The increased numbers of thymocyte subsets in rIL-7/HGFβ-treated allo-BMT recipients mice were maintained through day 75 post-BMT.** Lethally irradiated mice (BALB/c, 4-10 week old) were injected i.v. with 2 X10^6^ TCD-BM from B6 mice. Groups of mice were then injected i.p. with rIL-7/HGFβ (15 μg), or PBS at 2-day intervals from days 1 to 26 after BMT. The number of (A) total thymocytes, CD4 and CD8 DN, DP, CD4 SP, and CD8 SP thymocytes, and (B) donor-origin lineage^-^ c-kit^+^ IL-7Rα^-^ CD44^+^CD25^-^ ETPs was analyzed on day 75 after BMT. Means + S.D. are presented. The data are representative of 2 independent experiments with 5 mice per group. * P<0.05 compared with PBS-treated mice.
(TIF)

Figure S2 **rIL-7/HGFβ treatment increases the number of thymocytes subsets in a parent-F1 allo-BMT model.** Lethally irradiated B6C3F1 mice (4-10 week old) were injected i.v. with 2 X10^6^ TCD-BM from CD45.1^+^ B6 mice. Groups of mice were then injected i.p. with rIL-7/HGFβ (15 μg), or PBS at 2-day intervals from days 1 to 26 after BMT. The number of total thymocytes, CD4 and CD8 DN, DP, CD4 SP, and CD8 SP thymocytes was analyzed on day 30 after BMT. Means + S.D. are presented. The data are representative of 2 independent experiments with 5 mice per group. * P<0.05 compared with PBS-treated mice.
(TIF)

Figure S3 **Donor-origin T cells in rIL-7/HGFβ-treated BMT recipients have a diverse TCR repertoire.** Lethally irradiated BALB/c mice were injected with TCD-BM from B6 mice and treated with cytokines as in Figure 1. On day 75 after BMT, the expression of TCR Vβ families by donor-origin CD4^+^ and CD8^+^ T cells in the spleen was analyzed by flow cytometry. The results were compared with those of T cells from untreated non-BMT C57BL/6 and BALB/c mice. Data show mean percentages + SD from groups of 5 mice.
(TIF)
