# Supplementary figures and images for: Recombinant IL-7/HGFβ Hybrid Cytokine Enhances T Cell Recovery in Mice Following Allogeneic Bone Marrow Transplantation
Source: PLoS One. 2013 Dec 12;8(12):e82998. doi: 10.1371/journal.pone.0082998 (PMC3861470; doi:10.1371/journal.pone.0082998)

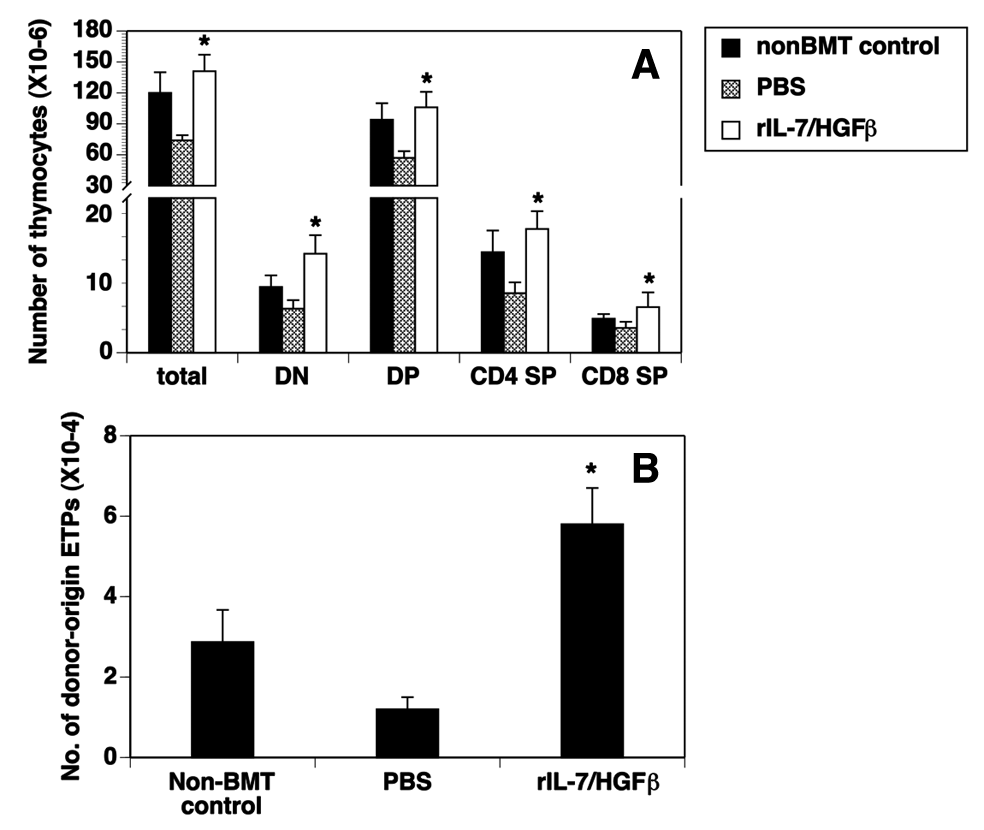

Supplement: Figure S1 — The increased numbers of thymocyte subsets in rIL-7/HGFβ-treated allo-BMT recipients mice were maintained through day 75 post-BMT. Lethally irradiated mice (BALB/c, 4-10 week old) were injected i.v. with 2 X106 TCD-BM from B6 mice. Groups of mice were then injected i.p. with rIL-7/HGFβ (15 μg), or PBS at 2-day intervals from days 1 to 26 after BMT. The number of (A) total thymocytes, CD4 and CD8 DN, DP, CD4 SP, and CD8 SP thymocytes, and (B) donor-origin lineage- c-kit+ IL-7Rα- CD44+CD25- ETPs was analyzed on day 75 after BMT. Means + S.D. are presented. The data are representative of 2 independent experiments with 5 mice per group. * P<0.05 compared with PBS-treated mice. (TIF) [file pone.0082998.s001.tif]

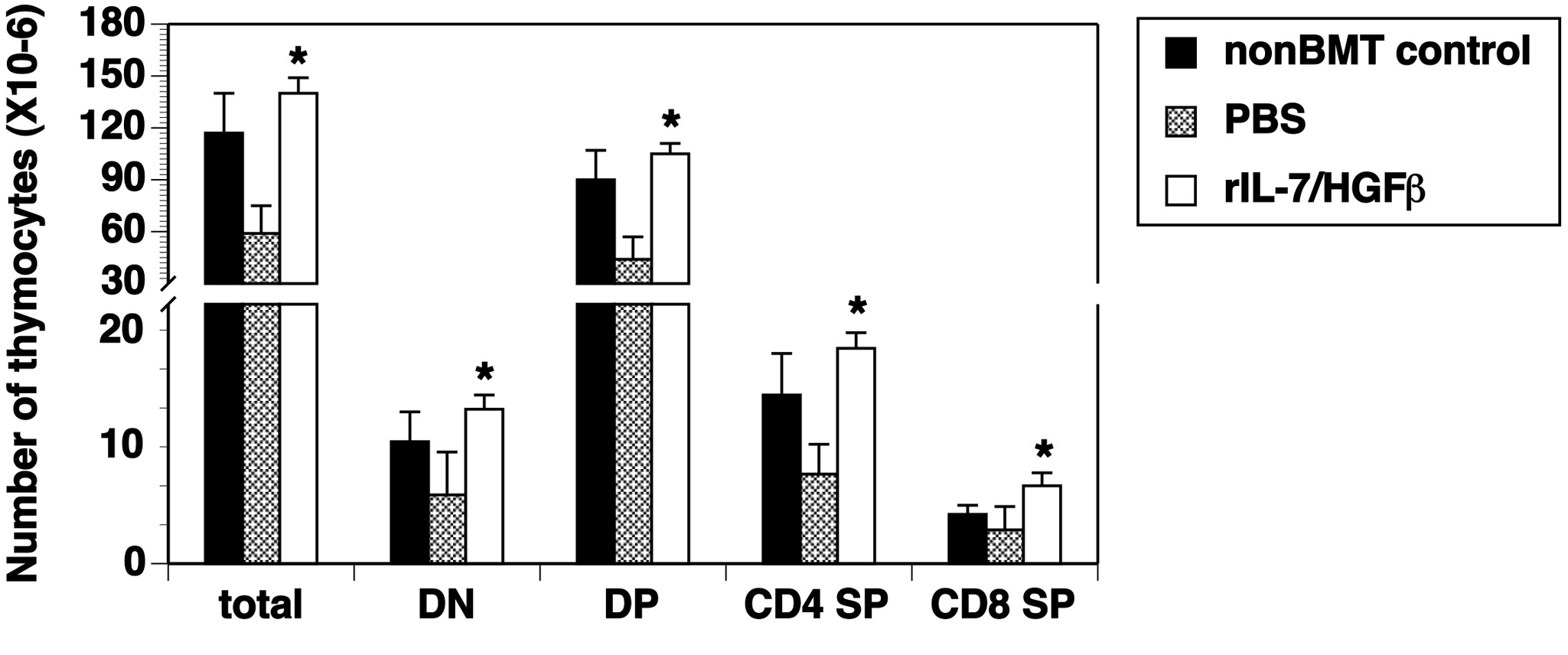

Supplement: Figure S2 — rIL-7/HGFβ treatment increases the number of thymocytes subsets in a parent-F1 allo-BMT model. Lethally irradiated B6C3F1 mice (4-10 week old) were injected i.v. with 2 X106 TCD-BM from CD45.1+ B6 mice. Groups of mice were then injected i.p. with rIL-7/HGFβ (15 μg), or PBS at 2-day intervals from days 1 to 26 after BMT. The number of total thymocytes, CD4 and CD8 DN, DP, CD4 SP, and CD8 SP thymocytes was analyzed on day 30 after BMT. Means + S.D. are presented. The data are representative of 2 independent experiments with 5 mice per group. * P<0.05 compared with PBS-treated mice. (TIF) [file pone.0082998.s002.tif]

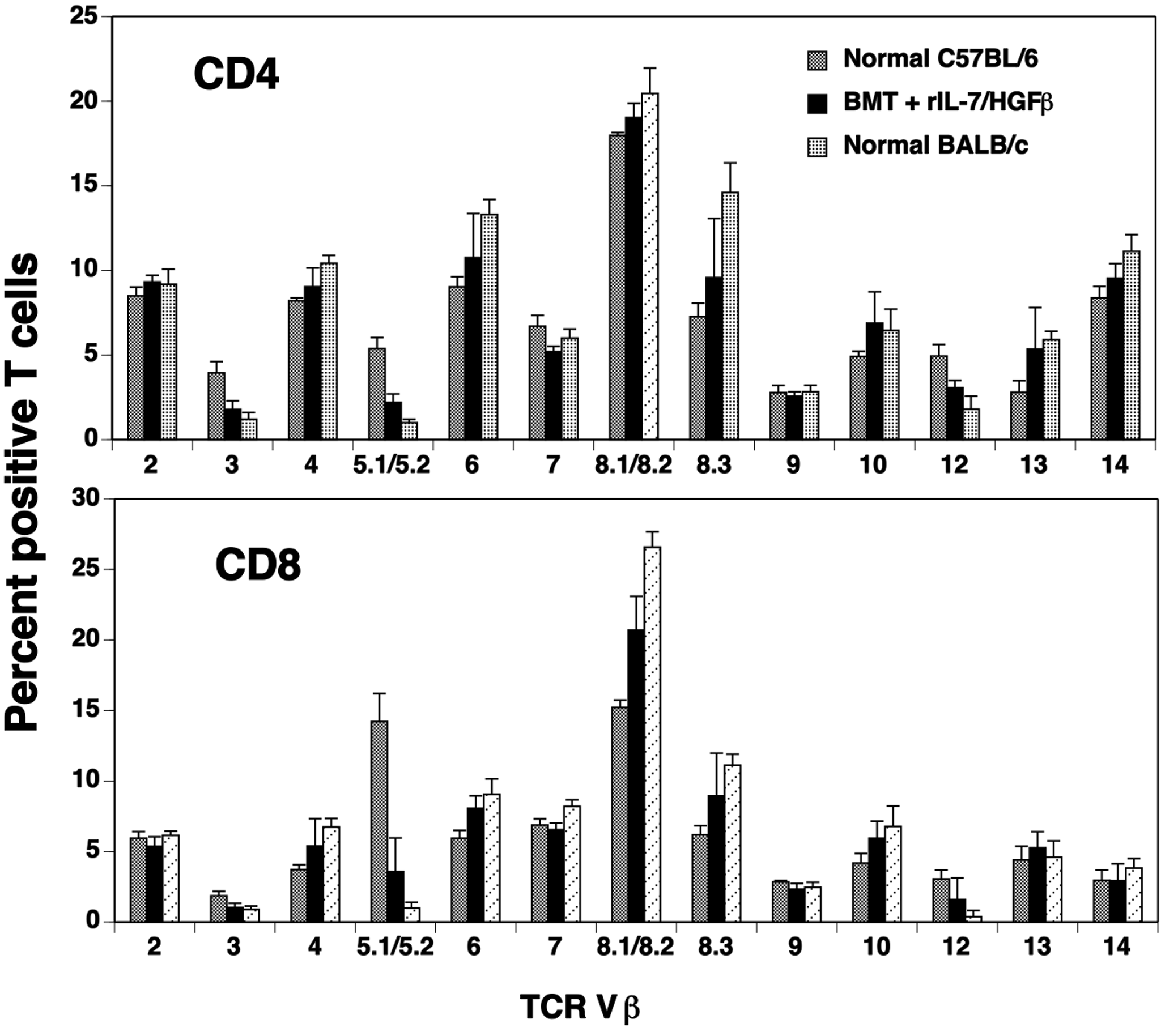

Supplement: Figure S3 — Donor-origin T cells in rIL-7/HGFβ-treated BMT recipients have a diverse TCR repertoire. Lethally irradiated BALB/c mice were injected with TCD-BM from B6 mice and treated with cytokines as in Figure 1. On day 75 after BMT, the expression of TCR Vβ families by donor-origin CD4+ and CD8+ T cells in the spleen was analyzed by flow cytometry. The results were compared with those of T cells from untreated non-BMT C57BL/6 and BALB/c mice. Data show mean percentages + SD from groups of 5 mice. (TIF) [file pone.0082998.s003.tif]
